# Prospective Study Examining Clinical Outcomes Associated with a Negative Pressure Wound Therapy System and Barker’s Vacuum Packing Technique

**DOI:** 10.1007/s00268-013-2080-z

**Published:** 2013-05-15

**Authors:** Michael L. Cheatham, Demetrios Demetriades, Timothy C. Fabian, Mark J. Kaplan, William S. Miles, Martin A. Schreiber, John B. Holcomb, Grant Bochicchio, Babak Sarani, Michael F. Rotondo

**Affiliations:** 1 Department of Surgical Education, Orlando Regional Medical Center, 86 West Underwood Street, Suite 201, Orlando, FL 32806 USA; 2 Los Angeles County/University of Southern California Medical Center, Los Angeles, CA USA; 3 University of Tennessee Health Science Center, Memphis, TN USA; 4 Albert Einstein Medical Center, Philadelphia, PA USA; 5 Carolinas Medical Center, Charlotte, NC USA; 6 Oregon Health Sciences University, Portland, OR USA; 7 University of Texas Health Science Center, Houston, TX USA; 8 University of Maryland, Baltimore, MD USA; 9 University of Pennsylvania, Pittsburgh, PA USA; 10 East Carolina University, Greenville, NC USA

## Abstract

**Background:**

The open abdomen has become a common procedure in the management of complex abdominal problems and has improved patient survival. The method of temporary abdominal closure (TAC) may play a role in patient outcome.

**Methods:**

A prospective, observational, open-label study was performed to evaluate two TAC techniques in surgical and trauma patients requiring open abdomen management: Barker’s vacuum-packing technique (BVPT) and the ABThera^TM^ open abdomen negative pressure therapy system (NPWT). Study endpoints were days to and rate of 30-day primary fascial closure (PFC) and 30-day all-cause mortality.

**Results:**

Altogether, 280 patients were enrolled from 20 study sites. Among them, 168 patients underwent at least 48 hours of consistent TAC therapy (111 NPWT, 57 BVPT). The two study groups were well matched demographically. Median days to PFC were 9 days for NPWT versus 12 days for BVPT (*p* = 0.12). The 30-day PFC rate was 69 % for NPWT and 51 % for BVPT (*p* = 0.03). The 30-day all-cause mortality was 14 % for NPWT and 30 % for BVPT (*p* = 0.01). Multivariate logistic regression analysis identified that patients treated with NPWT were significantly more likely to survive than the BVPT patients [odds ratio 3.17 (95 % confidence interval 1.22–8.26); *p* = 0.02] after controlling for age, severity of illness, and cumulative fluid administration.

**Conclusions:**

Active NPWT is associated with significantly higher 30-day PFC rates and lower 30-day all-cause mortality among patients who require an open abdomen for at least 48 h during treatment for critical illness.

## Introduction

The “open abdomen” (OA) and temporary abdominal closure (TAC) techniques have become valuable tools in the surgeon’s armamentarium. They are part of damage control strategies and are used in the treatment of abdominal sepsis and intraabdominal hypertension (IAH)/abdominal compartment syndrome (ACS). The carefully considered decision to abbreviate a patient’s laparotomy, leave the abdomen open, and apply a TAC in the presence of critical illness or intraabdominal catastrophe has been associated with improved patient survival [1–5]. Management of the OA in a patient with concomitant critical illness is challenging. It is associated with the potential for marked fluid loss, infection, visceral perforation, organ dysfunction, and death [6–13]. Prolonged abdominal decompression can result in intestinal adhesions, fascial retraction, loss of abdominal domain, formation of enteric fistulas, and development of massive incisional hernias requiring subsequent complex abdominal wall reconstruction [2, 7, 9, 12, 14–18]. Growing clinical experience has demonstrated that initial management defines the subsequent duration and complexity of the OA [2, 5, 19, 20].

The concept of TAC has steadily evolved over the past two decades, with a variety of techniques described. Modern TAC dressings may be classified into two broad classes based on their function: (1) passive visceral coverage (plastic silos and prosthetic meshes) and (2) negative pressure techniques that maintain abdominal wall integrity, preserve abdominal domain, and remove intraperitoneal fluid [16–18]. Mechanical abdominal wall retraction devices are increasingly being used in conjunction with TAC dressings to achieve the desired endpoint of primary fascial closure (PFC).

Clinical experience demonstrates that simple coverage of exposed viscera is no longer sufficient. Recent evidence suggests that the TAC technique chosen may moderate organ dysfunction and play a role in patient outcome [20, 21]. Animal studies suggest that active removal of cytokine-rich proinflammatory peritoneal fluid from the OA improves both pulmonary and renal function [21]. Human clinical trials have demonstrated that negative pressure wound therapy (NPWT) facilitates same-admission PFC [5, 22]. Improved resuscitation and earlier closure of the OA have been correlated with improved patient survival [2]. Active removal of cytokine-rich proinflammatory peritoneal fluid and early fascial closure should therefore be the goals of TAC therapy.

**Fig. 1:**
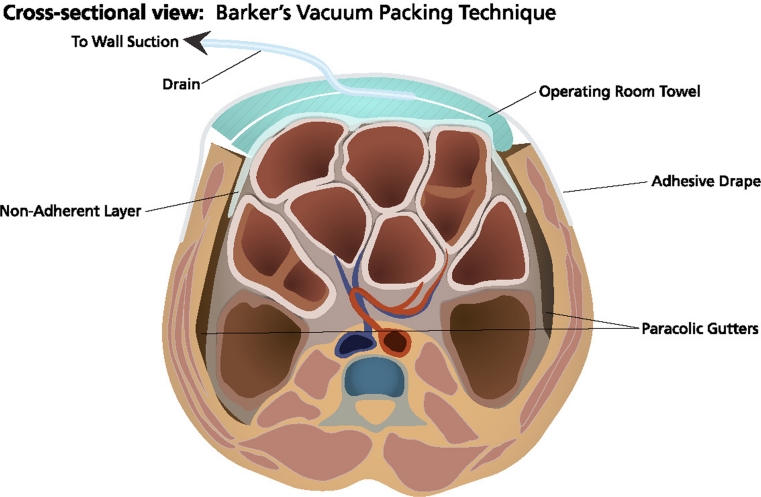
Barker’s vacuum-packing technique consists of a fenestrated, nonadherent polyethylene sheet placed over the viscera and covered with moist surgical towels or gauze. Two drains are placed over the towels/gauze. The wound is then sealed with an occlusive dressing and connected to wall suction (with permission from KCI Licensing, Inc.)

Of the various TAC methods, Barker’s vacuum-packing technique (BVPT) is the most commonly utilized due to its simplicity, cost, and availability of necessary materials in any operating room [16, 23]. The BVPT TAC typically consists of a fenestrated, nonadherent polyethylene sheet placed over the viscera and covered with either moist surgical towels or gauze. Two surgical drains are placed over the towels or gauze, the abdomen is sealed with a large adhesive dressing, and the drains are connected to variable levels of wall suction (Fig. 1). Local variations of this technique are common.

**Fig. 2:**
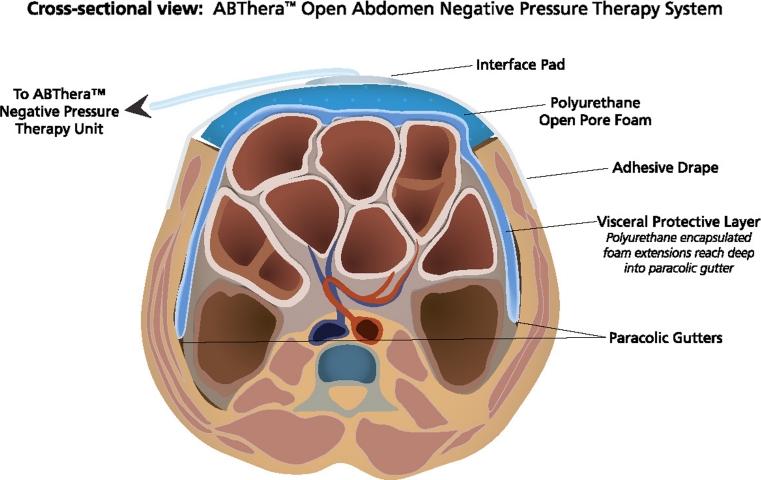
ABThera^TM^ open-abdomen negative-pressure therapy system, commercially available, is composed of a reusable negative pressure source (ABThera^TM^ pump), a visceral protective layer dressing composed of a nonadherent sheet with encapsulated foam struts, a sheet of polyurethane foam, an adherent elastic barrier layer, and a tubing set to connect the negative-pressure source to the dressing (with permission from KCI Licensing, Inc.)

NPWT techniques utilizing polyurethane foam and continuous suction are also widely employed to manage the OA. The ABThera^TM^ OA Negative Pressure Therapy System (KCI USA, San Antonio, TX, USA) utilizes a calibrated negative pressure source, a large visceral protective layer consisting of a polyurethane film-covered central foam structure with six arms of polyurethane foam extending from the center, two pieces of perforated polyurethane foam, and adhesive drapes (Fig. 2). The visceral protective layer is designed to separate the viscera from the abdominal wall (decreasing visceral adherence that may prevent subsequent abdominal closure) and remove peritoneal fluid from dependent areas of the abdomen such as the pelvis and paracolic gutters.

No clinical study has demonstrated superiority of one TAC technique over another. In an effort to evaluate clinical outcomes of two commonly used techniques, a prospective, multicenter, open-label, postmarketing evaluation comparing BVPT versus NPWT was performed.

## Methods

This was a prospective, multicenter, observational study with 20 participating trauma centers from across the United States. This study was registered with ClinicalTrials.gov (NCT01016353). The institutional review board (IRB) at each study site approved the study protocol with a waiver of informed consent given the emergent nature of abdominal decompression. Some study sites were required by their respective IRB to obtain subsequent informed consent to collect patient information. Recognizing that one TAC method or the other was considered “standard of care” at some centers and a prospective, randomized study design would not be approved by the IRB at other centers, the study sites were chosen with the intent that one-fourth of them would contribute BVPT patients only, one-fourth would contribute NPWT patients only, and one half would contribute patients receiving both treatments resulting in equal enrollment in each study arm.

An open-label, observational study design was chosen that allowed the surgeons at each study site to utilize the two TAC techniques and resuscitate patients as they deemed clinically appropriate. Although the intent of the study protocol was consistent use of one TAC method or the other, investigators were allowed to cross patients over to the other TAC technique at their discretion. Included in the study were surgical or trauma patients between 18 and 75 years of age who required either a BVPT or NPWT TAC following damage control laparotomy or treatment of either severe sepsis or IAH/ACS.

Patients were excluded if any of the following criteria were known to be present: pregnancy; active uncontrolled hemorrhage at the time of initial TAC placement; preexisting bleeding disorder; known allergy or hypersensitivity to polyvinyl, polyurethane, acrylic, or acrylic adhesive; preexisting abdominal fistulas; Child-Pugh liver dysfunction class C; body mass index > 40 kg/m^2^; New York Heart Association classification IV; chronic renal failure requiring dialysis; peritoneal dialysis/lavage; preexisting terminal illness; or significant abdominal wall defect as determined by the surgeon at the time of initial TAC placement.

Patients whose abdominal fascia and skin were not closed following laparotomy were defined as having an OA. “Surgical” patients underwent emergent, nontrauma procedures. “Trauma” patients were classified into “blunt” or “penetrating” categories based on the mechanism of the injury.

The primary outcome measure was time to, and the rate of, PFC at 30 days (defined as the act of closing the patient’s abdominal fascial defect by direct approximation of 100 % of its edges). The secondary outcome measure was 30-day all-cause mortality. Both the primary and secondary outcome measures were further analyzed according to the etiology of the patient’s OA (surgical vs. trauma). Other outcome measures evaluated included hours of mechanical ventilation, intensive care unit and hospital length of stay (LOS) and any TAC-related complications that occurred during the patient’s hospitalization.

Intraabdominal pressure (IAP) measurements were performed at the discretion of the treating physician(s). IAH was defined as sustained or repeated pathologic elevation in IAP ≥12 mmHg [14, 15]. ACS was defined as sustained IAP >20 mmHg (with or without an abdominal perfusion pressure <60 mmHg) associated with new organ dysfunction or failure [14, 15]. The severity of IAH was based on the highest IAP measured during the study period. Multiple organ dysfunction syndrome (MODS) was defined as the development of dysfunction within two or more of the following organ systems: pulmonary, renal, hepatic, cardiovascular.

Patient fluid resuscitation volumes were collected over the first 7 days of TAC therapy and stratified by type: crystalloid, packed red blood cells (pRBC), fresh frozen plasma (FFP). Peritoneal fluid drainage from the TAC dressings was similarly measured. Cumulative fluid balance over the first 7 days of TAC therapy was analyzed.

Standard demographic data were collected. Severity of illness was assessed using the Acute Physiology and Chronic Health Evaluation score (APACHE version III), the Sequential Organ Failure Assessment (SOFA) score, and the Injury Severity Score (ISS) (for trauma patients). As the majority of patients underwent laparotomy on the day of hospital admission, severity of illness scores were calculated using the first 24 h of clinical data following initial TAC placement. Patients were followed for 30 days from study enrollment and included patient outcome at hospital discharge (the “study period”).

The decision to perform damage control laparotomy, leave a patient’s abdomen open, and apply a TAC dressing at the time of initial laparotomy depends on many factors and varies from surgeon to surgeon. This variability results in two populations of patients: (1) those whose critical illness requires ongoing use of an OA with multiple TAC dressing changes and (2) those whose abdomen can be successfully closed at the time of the first TAC dressing change after damage control resuscitation has been achieved (typically 48 h after initial laparotomy). Patients with “early closures” have been demonstrated in previous studies to have a less severe illness, lower complication rate, and lower mortality than patients who require TAC therapy for >48 h [24–26]. The “all patients” population was defined as any patient who underwent either BVPT or NPWT as their initial TAC method. The “TAC ≥ 48 h” subpopulation was defined a priori as any patient who underwent either BVPT or NPWT as their initial TAC and received at least 48 h of consistent therapy. These patients were believed to represent the patient population of greatest clinical interest because of their increased complexity and illness severity. The “TAC < 48 h” subpopulation was defined as all patients who succumbed to their critical illness, achieved PFC, or received both TAC therapies within 48 h of the initial laparotomy.

The target sample size for this study was based on a parallel design and determined by an independent Data Monitoring Committee (DMC) after a planned interim assessment of the primary and secondary endpoints among the first 70 patients enrolled. At that time, the 30-day PFC rate was 65 % for BVPT and 81 % for NPWT. The DMC recommended that at least 271 evaluable patients would be required to confirm the 16 % difference in PFC rate between BVPT and NPWT with a power of 80 % and type I error rate of 5 %. The final maximum sample size was increased to 300 patients to allow for patient consent refusals, major protocol violations, and screening failures. Patient data were entered into a central web-based electronic database. Data queries requiring clarification were documented and returned to the study site for resolution.

Data were analyzed using Statistical Analysis System (SAS) software (version 9.1.3; SAS Institute, Cary, NC, USA) and are reported as the mean ± SD, median (interquartile range, or IQR), or percentage. Categoric data were analyzed using Fisher’s exact test. Continuous data were analyzed using analysis of variance or Wilcoxon’s rank-sum test, as appropriate. Median days to PFC, reported for all patients with failures censored at 30 days, was estimated using Kaplan–Meier curves, which were compared using the log-rank test. Multivariate logistic regression analyses were performed to determine which TAC technique was associated with 30-day PFC and survival utilizing the APACHE III score for severity of illness adjustment and incorporating all resuscitation variables determined to be significant by univariate analysis. Kaplan–Meier survival curve analysis and the log-rank test were utilized to compare each TAC technique with regard to survival over time. Statistical significance was defined as *p* < 0.05.

## Results

**Fig. 3:**
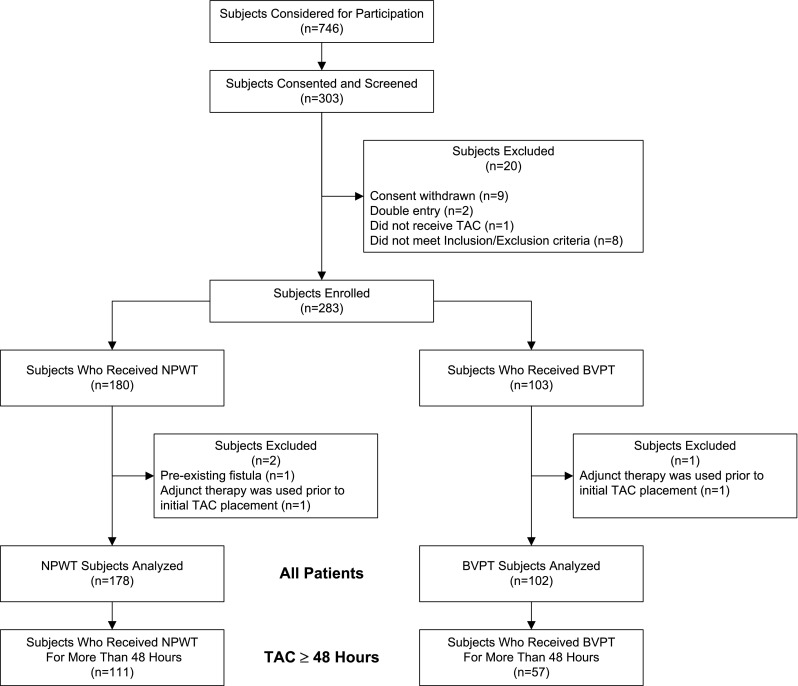
Consolidated Standards of Reporting Trials (CONSORT) statement. *BVPT* Barker’s vacuum packing technique, *NPWT* negative-pressure wound therapy, *TAC* temporary abdominal closure

Between November 2009 and January 2011, 20 study sites enrolled 283 patients who met the study inclusion and exclusion criteria (198 trauma, 85 emergency nontrauma) (Fig. 3). Three patients (2 NPWT, 1 BVPT) were subsequently excluded because of protocol violations. A larger proportion of NPWT TAC dressings were utilized than BVPT dressings (178 NPWT, 102 BVPT). Table 1 depicts the enrollment by study site for the 280 subjects. The demographics and severity of illness of the all patients population and the TAC < 48 h and TAC ≥ 48 h subpopulations are depicted in Table 2. Overall, surgical patients were older (51 ± 16 vs. 36 ± 14 years; *p* < 0.01), more likely to be female (42 vs. 15 %; *p* < 0.01), and had higher APACHE III scores (66 ± 23 vs. 54 ± 22; *p* < 0.01) than their trauma counterparts. There were no significant demographic differences between the all patients population and the TAC ≥ 48 h subpopulation. Patients in the TAC < 48 h subpopulation were less severely injured than those requiring TAC ≥ 48 h, as evidenced by lower APACHE III (*p* < 0.01) and SOFA (*p* < 0.01) scores. They were also more likely to have sustained penetrating trauma (*p* = 0.02). The number of TAC dressing changes in the all patients population and the TAC ≥ 48 h subpopulation were similar. The low number of patients with TAC dressing changes (9 NPWT, 4 BVPT) in the TAC < 48 h subpopulation reflects the high percentage of patients in this subpopulation (91 %) who achieved PFC within 48 h of initial laparotomy and did not require a TAC dressing change.

**Table 1:** Patient enrollment by study center

Center	All patients group		TAC < 48 h group		TAC ≥ 48 h group	
	NPWT	BVPT	NPWT	BVPT	NPWT	BVPT
NPWT only						
Carolinas MC	28	3	11	3	17	0
USC/LAC	28	1	8	0	20	1
Albert Einstein MC	12	0	5	0	7	0
University of Oklahoma MC	7	0	3	0	4	0
University of Kentucky MC	5	0	2	0	3	0
BVPT only						
University of Pennsylvania MC	1	12	0	9	1	3
UTHC—San Antonio	0	8	0	8	0	8
University of Louisville MC	0	7	0	4	0	3
Ben Taub General Hospital	0	4	0	1	0	3
University of Rochester MC	0	3	0	2	0	1
NPWT and BVPT						
Orlando Regional MC	19	13	5	7	14	6
University of Tennessee—Memphis MC	10	17	6	9	4	8
Oregon Health & Science Center	10	16	3	6	7	10
UTHC—Houston	25	0	16	0	9	0
University of Maryland Medical Center	4	10	1	1	3	9
Tulane University MC	14	0	5	0	9	0
Thomas Jefferson University MC	8	0	1	0	7	0
Scott & White MC	4	3	1	1	3	2
Shand’s Hospital/University of Florida	1	4	0	2	1	2
University of South Alabama MC	2	1	0	0	2	1

*NPWT* negative pressure wound therapy, *TAC* temporary abdominal closure, *BVPT* Barker’s vacuum pack therapy, *MC* Medical Center, *UTHC* University of Texas Healthsciences Center, *USC/LAC* University of Southern California/Los Angeles County

**Table 2:** Demographics

Parameter	All patients group	TAC < 48 h group	TAC ≥ 48 h group	p*
Patients (no.)	280	112	168	
Age (years)	40 ± 16	39 ± 16	41 ± 16	0.30
Sex (% male)	77	79	76	0.47
BMI (kg/m^2^)	28 ± 5 (280)	28 ± 5 (112)	29 ± 5 (168)	0.19
APACHE III	58 ± 23 (277)	51 ± 21 (109)	62 ± 24 (168)	<0.01
SOFA	8 ± 4 (274)	6 ± 3 (109)	8 ± 4 (165)	<0.01
ISS^a^	28 ± 14 (198)	26 ± 12 (88)	29 ± 15 (110)	0.36
TAC dressing changes	2 ± 3 (280)	0 ± 1 (112)	4 ± 3 (168)	<0.01
Injury				
Surgical	29 % (82)	21 % (24)	35 % (58)	0.02
Trauma				
Blunt	33 % (92)	32 % (36)	33 % (56)	0.90
Penetrating	38 % (106)	46 % (52)	32 % (54)	0.02

Numbers in parentheses are the number of patients

*BMI* body mass index, *APACHE III* Acute Physiology and Chronic Health Evaluation score, version III, *SOFA* Sequential Organ Failure Assessment, *ISS* Injury Severity Score

* Continuous variables were compared between TAC < 48 h and TAC ≥ 48 h using Wilcoxon’s rank sum test. Discrete variables were compared between TAC < 48 h and TAC ≥ 48 h with Fisher’s exact test

^a^Trauma patients only

The demographics of the three populations, stratified by TAC method, are listed in Table 3. In all three populations, the NPWT and BVPT groups were well matched. The indications for TAC were similar between the NPWT and BVPT groups. The NPWT and BVPT groups underwent a similar number of TAC dressing changes within all three populations.

**Table 3:** Demographics by the TAC method

Parameter	All patients group			TAC < 48 h group			TAC ≥ 48 h group		
	NPWT	BVPT	*p*	NPWT	BVPT	*p*	NPWT	BVPT	*p*
Patients (no.)	178	102		67	45		111	57	
Age (years)	40 ± 17	39 ± 16	0.87	38 ± 17	40 ± 15	0.43	42 ± 16	39 ± 16	0.47
Sex (% male)	80 % (142)	73 % (74)	0.18	81 % (54)	78 % (35)	0.81	79 % (88)	68 % (39)	0.13
Injury									
Surgical	29 % (52)	29 % (30)	1.00	21 % (14)	22 % (10)	1.00	34 % (38)	35 % (20)	1.00
Trauma									
Blunt	34 % (60)	31 % (32)	0.79	31 % (21)	33 % (15)	0.84	35 % (39)	30 % (17)	0.60
Penetrating	37 % (66)	39 % (40)	0.80	48 % (32)	44 % (20)	0.85	31 % (34)	35 % (20)	0.60
BMI (kg/m^2^)	28 ± 5 (178)	28 ± 5 (102)	0.89	28 ± 5 (67)	28 ± 6 (45)	0.63	29 ± 5 (111)	29 ± 5 (57)	0.69
APACHE III	57 ± 24 (176)	58 ± 23 (101)	0.61	50 ± 22 (65)	53 ± 21 (44)	0.32	61 ± 24 (111)	62 ± 24 (57)	0.76
SOFA	8 ± 4 (172)	7 ± 4 (102)	0.58	6 ± 3 (64)	6 ± 3 (45)	0.74	8 ± 4 (108)	8 ± 4 (57)	0.91
ISS^a^	28 ± 15 (126)	29 ± 12 (72)	0.26	26 ± 14 (53)	27 ± 11 (35)	0.37	29 ± 16 (73)	30 ± 14 (37)	0.42
Indications for TAC			0.28			0.05			0.73
ACS	8 % (14)	9 % (9)		2 % (1)	4 % (2)		12 % (13)	12 % (7)	
Damage control laparotomy	56 % (100)	63 % (64)		60 % (40)	62 % (28)		51 % (56)	57 % (34)	
Abdominal sepsis	22 % (39)	13 % (13)		21 % (14)	4 % (2)		23 % (25)	19 % (11)	
Surgeon suspected IAH	11 % (20)	15 % (15)		10 % (7)	24 % (11)		12 % (13)	7 % (4)	
Other^b^	3 % (5)	1 % (1)		8 % (5)	4 % (2)		4 % (4)	2 % (1)	
No. of dressing changes	3 ± 3 (178)	2 ± 3 (102)	0.34	0 % (67)	0 ± 1 (45)	0.5	4 ± 3 (111)	4 ± 3 (57)	0.83

Numbers in parentheses are the numbers of patients

*ACS* abdominal compartment syndrome, *IAH* intraabdominal hypertension

^a^Trauma patients only

^b^Dehiscence, fluid overload, retroperitoneal edema

The IAP was measured at the discretion of the patient’s surgeon, with 14 of 20 enrolling centers measuring IAP. In all, 97 of the 280 patients (67 NPWT, 30 BVPT) had their IAP measured at some point during the study, but inconsistent measurement (range 1–89 IAP measurements per patient) prevented meaningful assessment of predecompression or postdecompression values. The mean highest IAP measured during the study period was 17 ± 6 mmHg in the NPWT group and 19 ± 7 mmHg in the BVPT group (*p* = 0.17). Within the TAC ≥ 48 h subpopulation, the mean highest IAP was 18 ± 6 mmHg in the NPWT group (*n* = 49) and 19 ± 7 mmHg in the BVPT group (*n* = 17) (*p* = 0.41).

Within the TAC ≥ 48 h subpopulation, the study day 7 total fluid balance was significantly lower in the NPWT group compared to that in the BVPT group (16 ± 15 vs. 27 ± 28 L; *p* = 0.04), but total peritoneal fluid output did not differ (8 ± 7 vs. 10 ± 11 L; *p* = 0.44). pRBC transfusion did not differ between the NPWT and BVPT groups (4 ± 6 vs. 10 ± 11 L; *p* = 0.29). In contrast, FFP transfusion was significantly less in the NPWT group (3 ± 4 vs. 5 ± 6 L; *p* = 0.03). Perioperative blood product administration among NPWT versus BVPT patients achieved similar pRBC/FFP/PLT ratios: 1.0:1.2:0.2 versus 1.0:1.0:0.2.

Within the TAC ≥ 48 h surgical subpopulation, the hours of mechanical ventilation (196 ± 197 vs. 277 ± 189 h; *p* = 0.05), intensive care unit LOS (16 ± 13 vs. 21 ± 19 days; *p* = 0.16), and hospital LOS (27 ± 17 vs. 33 ± 23 days; *p* = 0.27) were shorter in NPWT patients, but the differences did not achieve statistical significance. Among trauma patients, there were no significant differences in hours of mechanical ventilation (350 ± 384 vs. 260 ± 190 h; *p* = 0.49) and intensive care unit LOS (22 ± 18 vs. 19 ± 17 days; *p* = 0.16). Hospital LOS did differ significantly between NPWT and BVPT trauma patients (43 ± 36 vs. 28 ± 22 days; *p* = 0.02). When stratified by severity of illness using APACHE III quartiles, there were no significant differences in hospital LOS between the NPWT and BVPT treatment groups. There were also no significant differences in complication rates between the NPWT and BVPT groups in either the all patients or TAC ≥ 48 h populations, although there was a trend toward less MODS in the NPWT group (Table 4).

**Table 4:** Complications

Complication	All patients group			TAC ≥ 48 h group		
	NPWT	BVPT	*p*	NPWT	BVPT	*p*
Abdominal abscess/infection	23 % (40)	26 % (26)	0.56	22 % (24)	25 % (14)	0.70
ACS	8 % (14)	8 % (8)	1.00	4 % (4)	2 % (1)	0.66
Abdominal wound dehiscence	2 % (3)	1 % (1)	1.00	2 % (2)	0 %	0.55
Anastomotic leak	4 % (7)	2 % (2)	0.49	5 % (5)	2 % (1)	0.67
Application site erosion	0 %	1 % (1)	0.36	0 %	2 % (1)	0.34
Coagulopathy	5 % (9)	5 % (5)	1.00	1 % (1)	2 % (1)	1.00
DVT	3 % (5)	4 % (4)	0.73	5 % (5)	2 % (1)	0.67
Fascial necrosis	2 % (4)	5 % (5)	0.29	4 % (4)	7 % (4)	0.45
GI ischemia/necrosis	7 % (13)	3 % (3)	0.18	10 % (11)	7 % (4)	0.78
Intestinal fistula	4 % (7)	4 % (4)	1.00	5 % (6)	7 % (4)	0.74
Intestinal obstruction	3 % (5)	0 %	0.16	3 % (3)	0 %	0.55
MODS	8 % (15)	16 % (16)	0.08	10 % (11)	19 % (11)	0.10
PE	1 % (1)	0 %	1.00	1 % (1)	0 %	0.34

Numbers in parentheses are the numbers of patients

*ACS* abdominal compartment syndrome, *DVT* deep vein thrombosis, *GI* gastrointestinal, *MODS* multiple organ dysfunction syndrome, *PE* pulmonary embolism

The primary and secondary outcome measures for all three study populations are shown in Table 5. The 30-day PFC rate was significantly higher for days to PFC and 30-day all-cause mortality significantly lower in the TAC < 48 h subpopulation than in the TAC ≥ 48 h subpopulation. Among patients requiring a TAC for at least 48 h, the 30-day PFC rate was significantly higher in patients treated with NPWT for all indications except penetrating trauma (Table 6). Median days to PFC were lower among NPWT patients than among BVPT patients, but the difference did not achieve statistical significance. Patient characteristics significantly associated with successful 30-day PFC in a univariate analysis included use of a NPWT TAC, lower APACHE III score, decreased FFP requirement, lower peritoneal fluid output, and lower cumulative fluid balance on study day 7 (Table 7).

**Table 5:** Patient outcome by study group

Outcome indicators	All patients group	TAC < 48 h group	TAC ≥ 48 h group	*p*
Patients (no.)	280	112	168	
30-Day PFC rate	74 % (208)	91 % (102)	63 % (106)	<0.01*
Interval to PFC (days)^a^	5 [3–12]	3 [2–3]	9 [5–15]	<0.01
30-Day all-cause mortality rate	15 % (41)	8 % (9)	19 % (32)	0.01*

Numbers in parentheses are the numbers of patients

*PFC* primary fascial closure, *IQR* interquartile range

*Fisher’s exact test

^a^Median [IQR]. The median days were estimated using Kaplan–Meier curves and were compared using the log-rank test

**Table 6:** 30-Day primary fascial closure

Parameter	All patients group			TAC < 48 h group			TAC ≥ 48 h group		
	NPWT	BVPT	*p*	NPWT	BVPT	*p*	NPWT	BVPT	*p*
Patients (no.)	178	102		67	45		111	57	
All patients	78 % (139)	68 % (69)	0.06	93 % (62)	89 % (40)	0.74	69 % (77)	51 % (29)	0.03
Injury									
Surgical	75 % (39)	53 % (16)	0.05	79 % (11)	80 % (8)	1.00	74 % (28)	40 % (8)	0.02
Trauma									
Blunt	75 % (45)	56 % (18)	0.10	91 % (19)	87 % (13)	1.00	67 % (26)	29 % (5)	0.02
Penetrating	83 % (55)	88 % (35)	0.59	100 % (32)	95 % (19)	0.38	68 % (23)	80 % (16)	0.37
Interval to PFC (days)^a^	4 (3–11)	5 (3–17)	0.29	3 (2–3)	3 (2–4)	0.19	9 (4–18)	12 (5–NC)	0.12

Numbers in parentheses are the numbers of patients

*NC* not calculable

Median [IQR]. The median days were estimated using Kaplan–Meier curves and were compared using the log-rank test

**Table 7:** 30-Day patient characteristics at study day 7 in the TAC ≥ 48 h subpopulation, by the PFC result

Characteristic	Successful PFC	Unsuccessful PFC	*p*
Patients (no.)	106	62	
Age (years)	40 ± 17	43 ± 16	0.20
APACHE III score	58 ± 23	68 ± 24	<0.01
SOFA score	8 ± 3	9 ± 4	0.11
ISS score^a^	28 ± 14	32 ± 16	0.29
Indications for TAC			0.40
ACS	9 % (9)	18 % (11)	
Damage control laparotomy	57 % (60)	48 % (30)	
Abdominal sepsis	22 % (23)	21 % (13)	
Surgeon suspected IAH	9 % (10)	11 % (7)	
Other^b^	4 % (4)	2 % (1)	
TAC method			0.02
NPWT	73 % (77)	55 % (34)	
BVPT	27 % (29)	45 % (28)	
Crystalloid (L)	21.8 ± 15.4	24.7 ± 18.9	0.71
pRBC (L)	4.7 ± 7.8	5.4 ± 8.2	0.86
FFP (L)	2.8 ± 4.3	4.6 ± 5.5	0.01
Peritoneal fluid output (L)	6.7 ± 6.9	11.7 ± 10.6	<0.01
Fluid balance (L)	15.6 ± 17.5	26.4 ± 24.6	<0.01

Numbers in parentheses are the numbers of patients

*pRBC* packed red blood cells, *FFP* fresh frozen plasma

^a^Trauma patients only

^b^Dehiscence, fluid overload, retroperitoneal edema

**Fig. 4:**
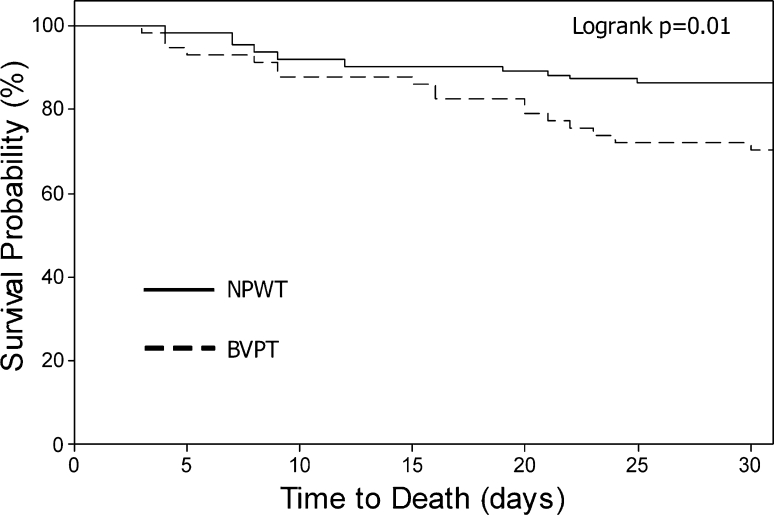
Kaplan–Meier plot of time to death for the TAC ≥ 48 h population

The 30-day all-cause mortality was significantly lower among patients treated for at least 48 h with a NPWT TAC (Table 8). When stratified by severity of illness, the mortality difference between NPWT and BVPT patients was most pronounced for patients in the middle two APACHE III quartiles. Patient characteristics significantly associated with decreased 30-day mortality in the univariate analysis included younger age; use of a NPWT TAC; lower APACHE III, SOFA, and ISS scores; successful 30-day PFC; decreased FFP requirement; lower peritoneal fluid output; and a lower cumulative fluid balance on study day 7 (Table 9). Kaplan–Meier survival curve analysis demonstrated that patients treated consistently for at least 48 h with NPWT were significantly more likely to survive 30 days than patients treated with BVPT (*p* = 0.01) (Fig. 4).

**Table 8:** 30-Day all-cause patient mortality rates

Parameter	All patients			TAC < 48 h			TAC ≥ 48 h		
	NPWT	BVPT	*p*	NPWT	BVPT	*p*	NPWT	BVPT	*p*
Patients (no.)	178	102		67	45		111	57	
All patients	12 % (21)	20 % (20)	0.08	9 % (6)	7 % (3)	0.74	14 % (15)	30 % (17)	0.01
Reason for laparotomy									
Surgical	17 % (9)	30 % (9)	0.27	21 % (3)	20 % (2)	1.00	16 % (6)	35 % (7)	0.11
Trauma									
Blunt	15 % (9)	25 % (8)	0.27	10 % (2)	0 % (0)	0.50	18 % (7)	47 % (8)	0.05
Penetrating	5% (3)	8 % (3)	0.67	3 % (1)	5 % (1)	1.00	6 % (2)	10 % (2)	0.62
APACHE III									
≤40	5 % (2)	4 % (1)	1.0	0 % (0)	0 % (0)	1.00	11 % (2)	8 % (1)	1.00
41–53	0 %	5 % (1)	0.31	0 % (0)	0 % (0)	1.00	0 % (0)	11 % (1)	0.23
54–72	14 % (6)	25 % (7)	0.35	25 % (3)	0 % (0)	0.22	10 % (3)	41 % (7)	0.02
≥73	30 % (13)	42 % (11)	0.43	30 % (3)	43 % (3)	0.64	30 % (10)	42 % (8)	0.55

Numbers in parentheses are the numbers of patients

**Table 9:** Survivor characteristics at study day 7 in the TAC ≥ 48 h subpopulation

Characteristic	Survivors	Nonsurvivors	*p*
Patients (no.)	136	32	
Age (years)	39 ± 16	50 ± 14	<0.01
APACHE III score	58 ± 23	76 ± 22	<0.01
SOFA score	8 ± 4	10 ± 3	<0.01
ISS score^a^	27 ± 14	40 ± 16	<0.01
Indications for TAC			0.44
ACS	10 % (13)	22 % (7)	
Damage control laparotomy	55 % (75)	47 % (15)	
Abdominal sepsis	22 % (30)	19 % (6)	
Surgeon suspected IAH	10 % (14)	9 % (3)	
Other^b^	3 % (4)	3 % (1)	
TAC method			0.01
NPWT	71 % (96)	47 % (15)	
BVPT	29 % (40)	53 % (17)	
30-Day PFC	71 % (97)	28 % (9)	<0.01
Crystalloid (L)	21.3 ± 14.6	29.5 ± 23.1	0.06
pRBC (L)	4.9 ± 8.1	5.2 ± 7.3	0.68
FFP (L)	3.1 ± 4.5	5.1 ± 5.9	0.01
Peritoneal fluid output (L)	7.5 ± 7.2	12.8 ± 12.7	0.02
Fluid balance (L)	15.9 ± 17.2	35.4 ± 27.5	<0.01

Numbers in parentheses are the numbers of patients

^a^Trauma patients only

^b^Dehiscence, fluid overload, retroperitoneal edema

30-day PFC rate varied widely by study site from 0 to 100 % with an overall rate of 74 %. Median days to PFC also varied widely by study site from 3 to 12 days. Multivariate logistic regression analysis was performed to adjust for confounding factors by inclusion of variables associated with successful 30-day PFC. Although there was a trend toward an increased likelihood of 30-day PFC among patients managed with NPWT in the TAC ≥ 48 h subpopulation [odd’s ratio 2.00 (0.98–4.08); *p* = 0.06], no variables in the model achieved significance in this analysis.

Multivariate logistic regression analysis was performed to adjust for potential confounding factors independently associated with 30-day survival including age, APACHE-III score (to adjust for severity of illness), and cumulative resuscitation fluids at study day seven. Patients treated with NPWT for at least 48 h were significantly more likely to survive compared to BVPT patients [odd’s ratio 3.17 (1.22–8.26); *p* = 0.02] (Table 10).

**Table 10:** Multivariate logistic regression analysis of 30-day survival controlling for age, severity of illness, and cumulative fluids at study day 7

Factor	Odds ratio	95 % CI	*p*
All patients			
NPWT	2.05	0.91–4.59	0.08
Age (years)	0.96	0.93–0.99	0.04
APACHE III (per point)	0.97	0.95–0.99	<0.01
Crystalloid (L)	0.96	0.94–0.99	<0.01
pRBCs (L)	1.27	1.07–1.51	<0.01
FFP (L)	0.72	0.59–0.88	<0.01
Peritoneal fluid output (L)	0.98	0.92–1.05	0.59
Total fluid output (L)	1.02	0.98–1.05	0.20
TAC ≥ 48 h			
NPWT	3.17	1.22–8.26	0.02
Age (years)	0.95	0.91–0.98	<0.01
APACHE III (per point)	0.99	0.97–1.01	0.23
Crystalloid (L)	0.97	0.94–0.99	0.01
pRBCs (L)	1.27	1.07–1.51	<0.01
FFP(L)	0.72	0.59–0.88	<0.01
Peritoneal fluid output (L)	0.99	0.93–1.07	0.87
Total fluid output (L)	1.01	0.97–1.05	0.54

Numbers in parentheses are the numbers of patients

*CI* confidence interval

## Discussion

Widespread use of damage-control principles for life-threatening abdominal conditions, recognition and treatment of IAH/ACS, and a new understanding of severe abdominal sepsis have resulted in an increase in the number of patients treated with an OA. The TAC method utilized for such OAs may play a significant role in patient outcome. Over the past decade, TAC has evolved from a simple, passive dressing of necessity to control massively distended viscera and organs into an active, therapeutic tool that potentially reduces elevated IAP, protects the abdomen from heat and fluid loss, removes proinflammatory cytokine-rich peritoneal fluid, and facilitates early PFC.

The success of OA management in many centers has been due, at least in part, to use of the “vacuum packing” technique described by Barker et al. [16]. This simple TAC method utilizes dressing supplies that are readily available in any operating room. Widely performed with numerous modifications, BVPT reduces elevated IAP by increasing the abdominal cavity volume, decreasing heat and fluid losses from exposed viscera, and controlling and quantifying fluid drainage from the OA.

The ABThera^TM^ system represents an advance in TAC therapy, performing the usual goals of expanding the abdominal cavity, protecting the viscera from heat and evaporative losses, and controlling and quantifying abdominal fluid. In addition, the large visceral protective layer can be positioned such that it actively removes potentially detrimental peritoneal fluid from deep within the abdomen while simultaneously decreasing visceral adherence to the abdominal wall. This study identifies that active NPWT is associated with significantly higher 30-day PFC rates and lower 30-day all-cause mortality among patients who require an OA for at least 48 h. Improved PFC rates have been demonstrated to correlate with significant increases in patient survival and decreases in hospital charges [2, 20]. Same-admission PFC, thereby avoiding an incisional hernia and the need for subsequent complex abdominal wall reconstruction, should be the goal in any patient who requires OA management.

This is the first study to demonstrate a survival advantage associated with a particular TAC technique. Although demographics, severity of illness, and indications for TAC were similar in the two treatment groups, the cumulative resuscitation requirement was significantly higher and more variable in the BVPT group. This difference may initially suggest a difference in fluid resuscitation strategy, but the increased fluid requirement may also be indicative of ongoing sepsis and inflammation in patients treated with a BVPT TAC, as suggested by the almost twofold higher rate of MODS among BVPT patients despite similar initial severity of illness. The difference in mortality rate between the NPWT and BVPT groups progressively widened over the first 30 days, consistent with late deaths due to MODS among the BVPT patients (Fig. 4). This raises the question of whether active NPWT more effectively removes detrimental cytokine-rich peritoneal fluid from the OA, reducing organ dysfunction and alleviating critical illness as suggested in previous animal studies. Clinical studies are currently underway to determine the efficacy of peritoneal cytokine removal by these two TAC techniques to further clarify this important question.

If improved peritoneal cytokine removal is the mechanism for the higher PFC and lower mortality rates witnessed in the NPWT treatment group, one might anticipate higher peritoneal fluid output in such patients. This study identified a nonsignificant trend toward higher peritoneal fluid output in the BVPT group. As total fluid intake is clinically correlated with peritoneal fluid output, we believe that the increased fluid requirement of the BVPT group may have driven the higher peritoneal fluid volumes seen and explain the lack of difference in peritoneal fluid removal between NPWT and BVPT patients. The clinical trials currently being performed should answer this question.

The safety of NPWT remains a concern for some surgeons. The incidence of critical complications such as development of ACS or an intestinal fistula during TAC therapy did not differ between the two study groups. The importance of serial IAP monitoring to diagnose recurrent ACS in patients with an OA and TAC cannot be overemphasized [27]. Recurrent ACS may occur with any TAC technique, especially in cases with active bleeding and clotted hemoperitoneum, which prevents effective removal of any intraperitoneal fluid. ACS, which may be related to worsening of the patient’s critical illness, is frequently attributed to an inadequate laparotomy incision or premature tightening of the TAC closure.

This observational study has several limitations. First, although the preferred methodology for an evaluation of these two TAC methods would be a randomized controlled trial, the IRB requirement to obtain informed consent prior to randomization of patients to either TAC technique during the initial application would have prevented study completion due to the lack of patient acuity and the frequent unavailability of family members to give consent. An open-label, observational study design with a waiver of informed consent was therefore chosen affording a “real-world” comparative effectiveness analysis of these two TAC techniques. This allowed surgeons to choose the TAC dressing that they believed was clinically indicated. Overall, surgeons elected to employ NPWT to a greater extent than BVPT, resulting in an uneven enrollment of subjects, although similar demographics and severity of illness, in each study arm. In this analysis, NPWT was independently associated with significantly better 30-day survival in patients who received at least 48 h of consistent TAC therapy. Sensitivity analyses of the study data stratified by high-enrolling centers and those that enrolled both NPWT and BVPT patients showed similar results regarding the survival benefit of NPWT. Second, we did not query the surgeons at the time of initial TAC placement as to the rationale behind their choice of TAC dressing. At many centers, this information would have been irrelevant as the standard of care was to use one TAC technique or the other. We cannot therefore determine whether patient-specific factors led to the surgeon’s choice of TAC method and the improved survival and PFC rate in the NPWT group. Third, it is difficult to evaluate the impact of NPWT on IAP in this patient population as IAP measurements were performed in only 35 % of the study patients. Goal-directed resuscitation using IAP measurements has been associated with improved patient survival [2]. Fourth, the source of the significant survival benefit of NPWT remains unclear. Patients who received TAC therapy for <48 h clearly differ in severity of illness and subsequent survival from those who received consistent TAC therapy for ≥48 h. We believe that at least some of these “early closure” patients may not have required an OA, and their inclusion in the study analyses would serve only to obscure the potential treatment benefits of the two TAC methods. The decreased number of patients in the a priori defined TAC ≥ 48 h subpopulation did result in reduced statistical power, which may be concealing causative factors for the treatment benefit identified that would be apparent in a larger study. Although our findings could be related to differences in resuscitation technique among study sites, the analyses performed thus far do not support this idea and suggest that a true treatment benefit exists. Fifth, marked variability in patient pathophysiology and differences in surgical management of the OA may have an impact on the success of OA closure. The large number of sites and observational nature of the study limited our ability to analyze these complex patterns of variability regarding both rate of, and days to, PFC. This may have obscured identification of factors independently predictive of successful 30-day PFC in the multivariate logistic regression analysis.

## Conclusions

A negative pressure therapy system was associated with significantly higher 30-day PFC rates and lower 30-day all-cause mortality among patients who required an OA for at least 48 h during treatment of their critical illness. Further investigation is required to determine the etiologies of these significant benefits in patient care and outcome.
